# Cytotoxic lanostane-type triterpenoids from the fruiting bodies of *Ganoderma lucidum* and their structure–activity relationships

**DOI:** 10.18632/oncotarget.14336

**Published:** 2016-12-28

**Authors:** Shaodan Chen, Xiangmin Li, Tianqiao Yong, Zhanggen Wang, Jiyan Su, Chunwei Jiao, Yizhen Xie, Burton B. Yang

**Affiliations:** ^1^ State Key Laboratory of Applied Microbiology Southern China, Guangdong Provincial Key Laboratory of Microbial Culture Collection and Application, Guangdong Institute of Microbiology, Guangzhou, China; ^2^ Yuewei Edible Fungi Technology Co. Ltd., Guangzhou, China; ^3^ College of Chinese Materia Medica, Guangzhou University of Traditional Chinese Medicine, Guangzhou, China; ^4^ Sunnybrook Research Institute, Sunnybrook Health Sciences Centre, Toronto, Canada; ^5^ Department of Laboratory Medicine and Pathobiology, University of Toronto, Toronto, Canada

**Keywords:** Ganoderma lucidum, lanostane-type triterpenoids, cytotoxicity, 3D-QSAR

## Abstract

We conducted a study of *Ganoderma lucidum* metabolites and isolated 35 lanostane-type triterpenoids, including 5 new ganoderols (**1**-**5**). By spectroscopy, we compared the structures of these compounds with known related compounds in this group. All of the isolated compounds were assayed for their effect against the human breast carcinoma cell line MDA-MB-231 and hepatocellular carcinoma cell line HepG2. Corresponding three-dimensional quantitative structure–activity relationship (3D-QSAR) models were built and analyzed using Discovery Studio. These results provide further evidence for anti-cancer constituents within *Ganoderma lucidum*, and may provide a theoretical foundation for designing novel therapeutic compounds.

## INTRODUCTION

Literature recording of *Ganoderma lucidum* (Fr.) P. Karst. (Ganodermataceae) was first found in *Sheng Nong's Herbal Classic* over two thousand years ago. Since then, fungi have been used as traditional Chinese medicines for the prevention or treatment of various chronic diseases [1, 2]. As research on the chemical constituents of these medicinal or edible fungi has increased, there is pre-clinical evidence for G. lucidum in an array of settings including cancer treatment [3–6], diabetic control [7–9], hepato-protection [10–13], antiviral treatment [14], and immune-modulation [15, 16].

In particular, *G. lucidum* in cancer research has become more prominent over the recent decades. Triterpenoids and polysaccharides are believed to form the pharmacodyamic material basis of the demonstrated anti-cancer effects. Lanostane-type triterpenoids are typical constituents of G. lucidum. Since the first triterpenoids (ganoderic acid A) were reported by Kubota in 1982, over 150 compounds have been isolated and reported in *G. lucidum* [17], with the number continually increasing. In order to search for bioactive anti-tumor metabolites, we launched a systematic study of the chemical constituents extracted from G. lucidum and other members of medicinal mushrooms [18–22]. In the present study, 35 triterpenoids were isolated, including 5 novel compounds. We then performed structural elucidation and cytotoxic assays using these compounds, and built 3D-QSAR models to predict anti-cancer activity.

## RESULTS AND DISCUSSION

**Figure 1:**
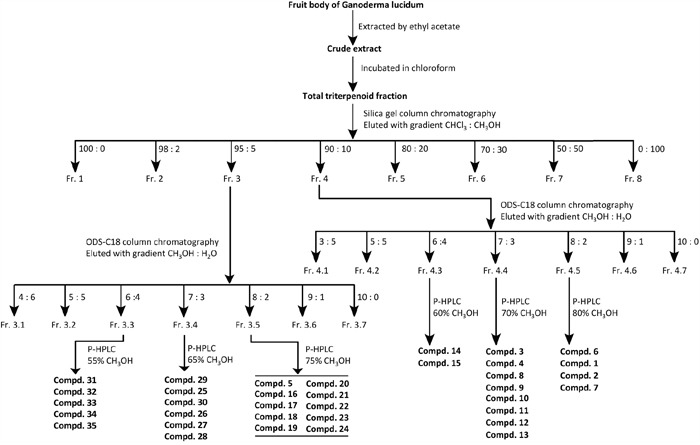
Purification procedure of compounds 1-35 from the fruit bodies of G. lucidum The purification procedure was started with the fruit body of *Ganoderma lucidum*. Each step is indicated by one arrow. Each Fraction (Fr.) was collected separately for further purification. Each compound (Compd.) was obtained in the final step and stored at -20°C for further analysis.

**Figure 2:**
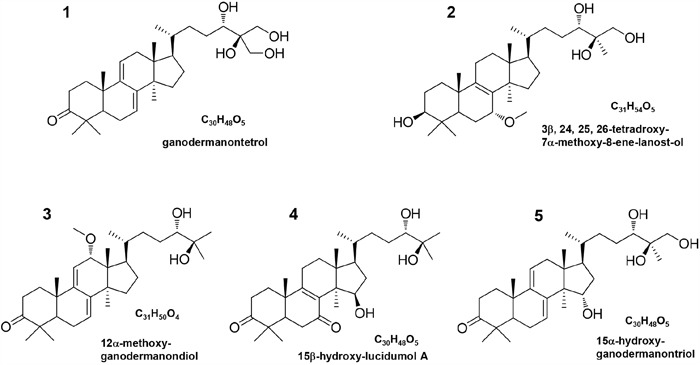
Structures and names of new compounds 1–5

Repeated column chromatography of the CHCl_3_-soluble fraction from the ethyl acetate extract of the fruiting bodies of *Ganoderma lucidum* resulted in the isolation of **30** known compounds (Compounds **6**–**35**, Figure 1) and five new compounds (Compounds **1–5**, Figure 2). The known compounds were identified as ganoderiol D (**6**) [23], ganoderiol F (**7**) [23], ganoderiol B (**8**) [14], ganoderiol E (**9**) [23], ganoderic acid β (**10**) [24], ganoderic acid A (**11**) [25], ganoderic acid B (**12**) [25], ganoderic acid C (**13**) [26], ganoderic acid D2 (**14**) [26], 12*β*-hydroxy-3,7,11,15,23-pentaoxo-lanosta-8-en-26-oic acid (**15**) [27], ganodermanontriol (**16**) [28], 3, 7, 11-trione-24(*S*), 25-dihydroxy-lanosta-8-ene (**17**) [29], ganoderitriol M (**18**) [30], lucidumol A (**19**) [14], lucidadiol (**20**) [31], ganoderiol A (**21**) [32], 24*R*, 25*S*, 26-trihydroxy-lanosta-7,9(11)-dien-3-one (**22**) [33], lucidumol B (**23**) [28], ganodermanondiol (**24**) [28], lucidenic acid A (**25**) [34], ganolucidic acid A (**26**) [35], ganoderic acid J (**27**) [36], methyl lucidenate A (**28**) [37], ganoderic acid E (**29**) [38], ganoderenic acid d (**30**) [27], ganoderic acid C2 (**31**) [37], ganoderic acid F (**32**) [38], ganoderic acid G (**33**) [27], ganoderic acid H (**34**) [38] and ganoderic acid AM (**35**) [39].

**Figure 3:**
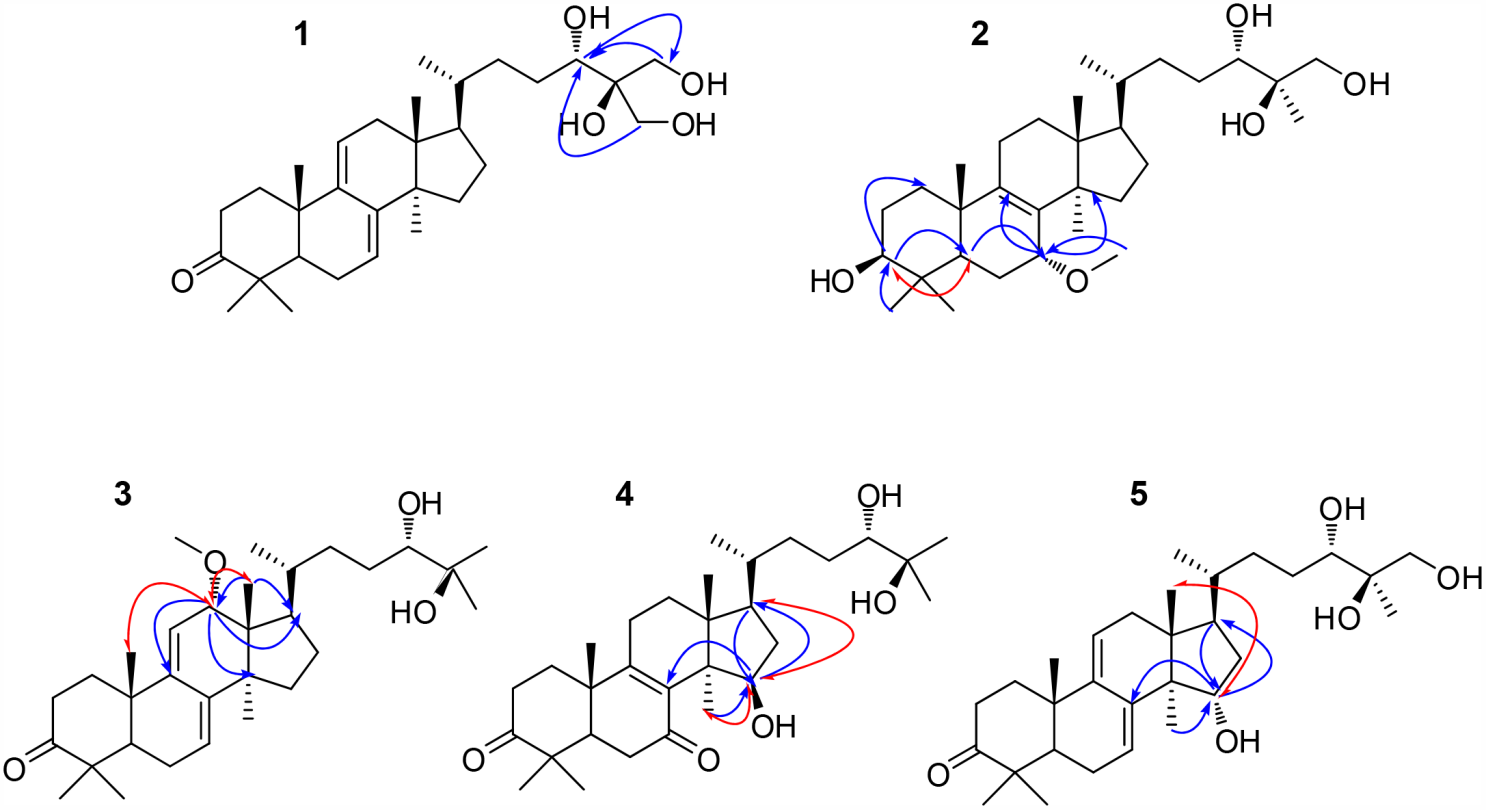
Key HMBC (
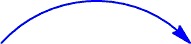
) and ROESY(
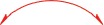
) correlations of compounds 1−5

Compound **1** was isolated as a white powder, [α]_D_^25^ = +13.4 (c 0.20, CHCl_3_). Its HR-ESI-MS spectrum gave a molecular ion peak at *m/z* 484.3187 corresponding to the molecular formula C_30_H_48_O_5_. The ^1^H NMR spectrum of compound **1** (Table 1) was indicative of five tertiary methyls (*δ*_H_ 1.21, 1.12, 1.09, 0.86, 0.64) and a secondary methyl [*δ*_H_ 0.91 (d, *J* = 6.0 Hz)] group, two oxygen bearing methylene signal [*δ*_H_ 3.45 (m) and 3.52 (m)], and one oxygen bearing methine signal [*δ*_H_ 3.45 (m)], two olefinic protons [*δ*_H_ 5.51 (dd, *J* = 6.0, 1.8 Hz) and 5.40 (dd, *J* = 6.0, 1.8 Hz)], respectively. The ^13^C NMR (Table 2) and DEPT-135 spectra exhibited the presence of 30 carbons due to six methyls, ten methylenes including two oxymethylenes, six methines including an oxymethine and eight quaternary carbons including a keto carbonyl. Comparison of these spectroscopic data with those of ganodermanontriol (**16**) [28], it was suggested that the skeleton moiety of compound **1** was almost the same except that one methyl in the side-chain was oxidized to hydroxymethyl. The location of the hydroxymethyl group was confirmed by the analysis of its HMBC spectrum. In the HMBC spectrum, there were correlations between the proton signals at *δ*_H_ 3.45 (H_2_-26, m), 3.52 (H_2_-27, m) and the carbon resonance at *δ*_C_ 70.9 (C-24), and between the proton signals at *δ*_H_ 3.45 (H-24, m) and the carbon signal at *δ*_C_ 62.2 (C-26) and 62.8 (C-27) (Figure 3). Thus, the structure of compound **1** was determined to be ganodermanontetrol. As compound **1** was derived from ganodermanontriol (**16**), it would have the same absolute configuration with ganodermanontriol. Consequently, the absolute configuration of C-24 in compound **1** was assigned as 24*S*.

**Table 1:** ^1^H NMR spectral data of compounds 1−5*^a^* in CDCl_3_

	1	2	3	4	5
1	2.37 (1H, m) *^a^*, 1.80 (1H, m)	1.79 (2H, m)	2.10 (1H, m), 1.75 (1H, m)	2.10 (1H, m), 1.75 (1H, m)	2.35 (1H,m), 1.80 (1H,m)
2	2.75 (1H, m), 2.35 (1H, m)	2.72 (1H, m), 2.23 (1H,m)	2.63 (2H, m)	2.63 (2H, m)	2.70 (2H,m)
3		3.25 (1H,m)			
4					
5	1.56 (1H, m)	1.65 (1H, m)	1.47 (1H, m)	1.47 (1H, m)	1.57 (1H,m)
6	2.10 (2H, m)	2.40 (2H, m)	2.42 (2H, m)	2.42 (2H, m)	2.08 (2H,m)
7	5.51 (1H, dd, 6.0,1.8)	4.12 (1H, br.s)			5.52 (1H,dd, 6.0,1.8)
8					
9					
10					
11	5.40 (1H,dd, 6.0,1.8)	2.17 (2H, m)	2.31 (2H, m)	2.31 (2H, m)	5.39 (1H,dd, 6.0,1.8)
12	2.19 (2H, m)	1.89 (2H, m)	4.37 (1H, m)	1.79 (2H, m)	2.20 (2H,m)
13		—			
14		—			
15	1.68 (1H, m), 1.41 (1H, m)	1.57 (2H, m)	1.79 (2H, m)	4.37 (1H, m)	4.28 (1H, m)
16	2.05 (1H, m), 1.37 (1H, m)	2.00 (2H, m),	2.35 (2H, m)	2.35 (2H, m)	2.05 (2H,m)
17	1.60 (1H, m)	1.58 (1H, m)	1.59 (1H, m)	1.59 (1H, m)	1.60 (1H,m)
18	0.64 (3H, s)	0.66 (3H, s)	0.62 (3H, s)	0.62 (3H, s)	0.62 (3H,s)
19	1.09 (3H, s)	1.19 (3H, s)	1.32 (3H, s)	1.32 (3H, s)	1.09 (3H,s)
20	1.50 (1H, m)	1.47 (1H, m)	1.45 (1H, m)	1.45 (1H, m)	1.49 (1H,m)
21	0.91 (3H, d, 6.0)	0.94 (3H, d, 6.0)	0.93 (3H,d, 5.4)	0.93 (3H,d, 5.4)	0.91 (3H,d, 6.0)
22	1.58 (1H, m), 1.04 (1H, m)	1.49 (1H, m), 1.05 (1H, m)	1.58 (1H, m), 1.04 (1H, m)	1.58 (1H, m), 1.04 (1H, m)	1.55 (1H,m), 1.05 (1H,m)
23	1.72 (2H, m),	1.67 (2H, m),	1.78 (1H, m), 1.53 (1H, m)	1.78 (1H, m), 1.53 (1H, m)	1.78 (1H,m), 1.53 (1H,m)
24	3.45 (1H, m)	3.52 (1H, m)	3.29 (1H, d, 9.6)	3.29 (1H, d, 9.6)	3.45 (1H,m)
25					
26	3.45 (2H, m)	3.82 (2H, d, 11.4)	1.12 (3H, s)	1.12 (3H, s)	3.82 (2H, d, 11.4)
27	3.52 (2H, m)	1.20 (3H, s)	1.22 (3H, s)	1.22 (3H, s)	1.20 (3H,s)
28	0.86 (3H, s)	0.83 (3H, s)	1.10 (3H, s)	1.10 (3H, s)	0.86 (3H,s)
29	1.12 (3H, s)	1.06 (3H, s)	1.09 (3H, s)	1.09 (3H, s)	1.12 (3H,s)
30	1.21 (3H, s)	1.12 (3H, s)	1.16 (3H, s)	1.16 (3H, s)	1.21 (3H,s)
-OCH_3_		3.38 (3H, s)	3.34 (1H, s)		

*^a^* means multiplet or overlapped with other signals.

**Table 2:** ^13^C NMR spectral data of compounds 1−5 in CDCl_3_

	1	2	3	4	5
1	36.6	35.0	36.5	35.4	36.6
2	34.9	27.7	34.7	34.4	34.8
3	216.6	78.5	216.4	215.0	216.9
4	47.5	38.6	47.4	47.2	47.4
5	50.3	48.2	50.4	49.7	50.5
6	23.8	24.0	23.5	37.2	23.7
7	119.8	76.1	124.1	198.0	119.8
8	142.9	135.7	142.2	139.6	142.9
9	144.5	139.5	149.9	162.8	144.5
10	37.3	37.5	38.5	39.3	37.3
11	117.3	21.5	118.2	23.8	117.3
12	37.8	30.2	79.2	30.2	37.8
13	43.8	45.0	49.0	45.0	43.8
14	50.7	49.7	52.9	51.5	52.1
15	31.5	31.8	31.0	73.4	74.2
16	27.9	28.5	27.0	39.7	40.3
17	51.0	49.6	52.5	50.5	50.2
18	15.7	16.3	11.7	15.9	15.7
19	22.5	17.5	21.7	17.9	22.5
20	36.5	36.4	35.6	36.2	36.4
21	18.6	18.5	18.4	18.4	18.6
22	31.4	34.5	34.7	34.7	34.4
23	28.9	28.8	28.7	28.7	28.9
24	70.9	79.5	79.7	79.7	79.3
25	74.9	73.5	74.5	73.7	73.8
26	62.2	67.7	26.5	26.5	67.9
27	62.8	22.2	23.2	23.2	22.0
28	25.4	27.4	27.6	27.6	25.4
29	25.3	15.4	20.2	20.2	21.2
30	21.2	25.2	25.5	25.5	25.3
-OCH_3_		55.5	55.8		

Compound **2** was isolated as a white amorphous powder with optical rotation of +22.6 (c 0.20; CHCl_3_). The molecular formula of compound **2** was found to be C_31_H_54_O_5_ on the basis of a molecular ion peak at an *m/z* value of 505.3882 [M - H]^-^ in the HR-ESI-MS. The ^1^H NMR spectrum of compound **2** (Table 1) displayed signals for six tertiary methyls at *δ*_H_ (1.20, 1.19, 1.12, 1.06, 0.83, 0.66), a secondary methyl at [*δ*_H_ 0.94 (d, *J* = 6.0 Hz)], an methoxyl at *δ*_H_ 3.38 (3H, s), one oxygen bearing methylene signal at *δ*_H_ 3.82 (2H, d, *J* = 11.4 Hz), and three oxygen bearing methine signal [*δ*_H_ 4.12 (1H, br.s), 3.52 (1H, m), 3.25 (1H, m)]. The ^13^C NMR spectrum (Table 2), combined with the DEPT-135 data, showed that compound **2** had 31 carbon signals consisting of eight methyls, ten methylenes, six methines and seven quaternary carbons. Comparison of the NMR data of compound **2** with those of ganoderiol D (**6**) [23] indicated that they were closely related to their structures, except of two keto carbonyl group at C-3 and C-7 in ganoderiol D being replaced by a hydroxyl and a methoxyl groups in compound **2**, respectively. The difference was confirmed by the significant change of the chemical shift value for C-3 and C-7 from *δ*_C_ 214.6 and 198.1 in ganoderiol D to *δ*_C_ 78.5 and 76.1 in compound **2**, which was consistent with its molecular formula. The linkage position of the hydroxyl at C-3 was further supported by significant HMBC correlations from *δ*_H_ 3.25 (H-3) to *δ*_C_ 35.0 (C-1) and 48.2 (C-5), from *δ*_H_ 0.83 (H-28) and 1.06 (H-29) to *δ*_C_ 78.5 (C-3) (Figure 3). In addition, the HMBC correlations between *δ*_H_ 4.12 (H-7) to *δ*_C_ 139.5 (C-9) and *δ*_C_ 49.7 (C-14), *δ*_H_ 1.65 (H-5) to *δ*_C_ 76.1 (C-7), and between *δ*_H_ 3.38 (H-31) to *δ*_C_ 76.1 (C-7) indicated the presence of a methoxyl groups at C-7. The configuration of compound **2** was determined by analyzing the NOESY spectrum (Figure 3). Key ROESY correlations were observed between H-3 and H-5, indicating that H-3 was on the *α*-orientation same as H-5. H-7 resonated as a broad singlet, indicating the presence of *β*-orientation. On the basis of the above evidence, the structure of compound **2** was identified as 3*β*, 24*S*, 25*R*, 26-tetradroxy-7*α*-methoxy-8-ene-lanost-ol.

Compound **3** was isolated as a white amorphous powder, [α]_D_^25^ = 24.8 (c 0.20, CHCl_3_). The molecular formula of compound **3** was found to be C_31_H_50_O_4_ on the basis of a molecular ion peak at an *m/z* value of 485.3642 [M - H] in the HR-ESI-MS. The ^1^H and ^13^C NMR spectra displayed were similar to those of compounds **1** and **16**. The significant difference was the presence of a methoxyl group connecting to C-12 in compound **3** and a hydroxymethyl at C-26 in compound **16** was deoxidated to a methyl in compound **3**. Location of this methoxyl (C-12) was assigned on the basis of the HMBC correlations from *δ*_H_ 0.62 (H-18) to *δ*_C_ 79.2 (C-12) and 52.5 (C-17) and the correlations from *δ*_H_ 4.37 (H-12) to δ_C_ 149.9 (C-9), 52.9 (C-14) and 52.5 (C-17). The relative configurations of H-7 and H-12 were assigned as *α*- and *β*-orientation, respectively, on the basis of ROESY correlations of H-12 with H3-18 and H3-19 (*δ*_H_ 1.32, s) (Figure 3). Therefore, the structure of compound **3** was elucidated as 12*α*-methoxy-ganodermanondiol.

The molecular formula of compound **4** was established as C_30_H_48_O_5_ by HR-ESI-MS data. Its ^1^H and ^13^C NMR spectroscopic data (Tables 1 and 2) revealed a similar structure to lucidumol A (**19**) [14] except for an extra hydroxyl [*δ*_H_ 4.37 (m); *δ*_C_ 73.4]. The location of a hydroxy group at C-15 was confirmed by the HMBC correlations from *δ*_H_ 1.16 (H_3_-30) to C-15, from *δ*_H_ 1.59 (H-17) to C-15, and from H-15 to *δ*_C_ 139.6 (C-8), 50.5 (C-17) and 25.5 (C-30) (Figure 3). The relative configuration of H-15 was assigned as *α*-orientation by the ROESY correlations of H-15 with H-17 and H_3_-30 (Figure 3). Thus, compound **4** was identified as 15*β*-hydroxy-lucidumol A.

Compound **5** was assigned the molecular formula C_30_H_48_O_5_ by HR-ESI-MS data. A detailed comparison of ^1^H and ^13^C NMR spectral data (Tables 1 and 2) between compound **5** and compound **16** indicated that compound **5** was a hydroxylated derivative of compound **16**. The hydroxyl moiety was deduced from signals due to one more oxygen bearing methine group (*δ*_H_ 4.28, m; *δ*_C_ 74.2). The HMBC correlations from *δ*_H_ 1.21 (H_3_-30) to C-15, from *δ*_H_ 1.60 (H-17) to C-15, and from H-15 to *δ*_C_ 142.9 (C-8), 50.2 (C-17) and 25.3 (C-30) (Figure 3) confirmed the hydroxyl at C-15. The *β*-orientation of H-15 was assigned by the ROESY correlations of H-15 with *δ*_H_ 0.62 (H_3_-18) (Figure 3). Thus, compound **5** was identified as 15*α*-hydroxy-ganodermanontriol.

It has been reported that triterpenoids possess cytotoxic activity on human cancer cells. We analyzed the effect of the isolated triterpenoids on human breast cancer cells MDA-MB-231 and hepatocellular cancer cells HepG2, and the results were summarized in Table 3. Among the compounds examined, compounds **1**-**3** were highly cytotoxic in both types of cancers. Compounds **4, 7, 20** and **24** exhibited moderate cytotoxicity. The rest compounds showed weak inhibition against the cancer cells. Since all compounds displayed similar effects on both cancer cells, we performed cell survival assay using compounds **1**-**4, 8, 17, 20, 22** and **24** on MDA-MB-231 cells. Compounds **3** and **20** appeared to produce significantly stronger effect on the viability of MDA-MB-231 cells (Table 4).

**Table 3:** Cytotoxicity of compounds 1–35 against human breast carcinoma cell line MDA-MB-231 and human hepatocellular carcinoma cell line HepG2*^a^*

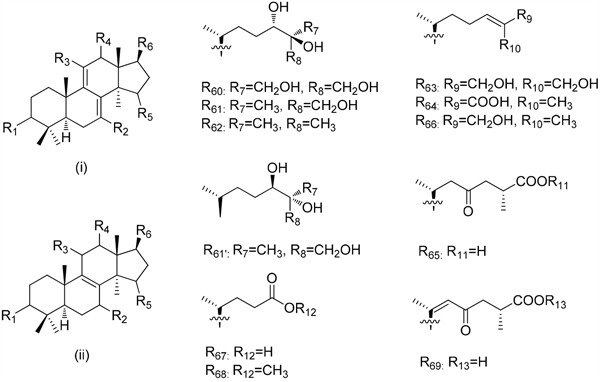									
**Compounds**	**Unit**	**R_1_**	**R_2_**	**R_3_**	**R_4_**	**R_5_**	**R_6_**	**IC_50_**	
								**MDA-MB-231**	**HepG2**
**1**	i	=O	H	H	H	H	R60	53.4 ± 9.9	43.7 ± 1.4
**2**	ii	β-OH	α-OCH_3_	H	H	H	R61	35.9 ± 0.4	39.3 ± 1.3
**3**	i	=O	H	H	α-OCH_3_	H	R62	21.2 ± 0.7	41.5 ± 3.2
**4**	ii	=O	=O	H	H	β-OH	R62	75.7 ± 1.9	82.6 ± 5.8
**5**	i	=O	H	H	H	α-OH	R61	112.1 ± 2.1	56.8 ± 1.7
**6**	ii	=O	=O	H	H	H	R61	102.3 ± 2.3	131.4 ± 3.1
**7**	i	=O	H	H	H	H	R63	77.0 ± 1.6	81.5 ± 2.5
**8**	i	=O	H	H	H	α-OH	R63	95.6 ± 2.1	92.6 ± 3.3
**9**	ii	β-OH	=O	H	H	H	R63	158.7 ± 3.2	>250
**10**	ii	β-OH	β-OH	=O	H	=O	R64	>250	>250
**11**	ii	=O	β-OH	=O	H	α-OH	R65	>250	>250
**12**	ii	β-OH	β-OH	=O	H	=O	R65	>250	>250
**13**	ii	=O	β-OH	=O	H	=O	R65	>250	>250
**14**	ii	=O	β-OH	=O	β-OH	=O	R65	>250	>250
**15**	ii	=O	=O	=O	β-OH	=O	R65	>250	>250
**16**	i	=O	H	H	H	H	R61	>250	>250
**17**	ii	=O	=O	=O	H	H	R62	92.5 ± 2.4	68.5 ± 2.2
**18**	ii	β-OH	=O	H	H	H	R62	>250	>250
**19**	ii	=O	=O	H	H	H	R62	105.8 ± 2.4	89.6 ± 2.0
**20**	ii	β-OH	=O	H	H	H	R66	76.6 ± 2.3	80.7 ± 1.1
**21**	i	β-OH	H	H	H	H	R61	158.0 ± 2.8	97.8 ± 1.7
**22**	i	=O	H	H	H	H	R6′	105.8 ± 3.1	169.4 ± 3.5
**23**	i	β-OH	H	H	H	H	R62	163.5 ± 2.9	146.7 ± 5.6
**24**	i	=O	H	H	H	H	R62	76.4 ± 0.9	51.8 ±1.1
**25**	ii	=O	β-OH	=O	H	=O	R67	>250	>250
**26**	ii	=O	H	=O	H	α-OH	R65	>250	>250
**27**	ii	=O	=O	=O	H	α-OH	R65	>250	>250
**28**	ii	=O	β-OH	=O	H	=O	R68	158.7 ± 1.7	>250
**29**	ii	=O	=O	=O	H	=O	R65	>250	>250
**30**	ii	=O	β-OH	=O	H	=O	R69	>250	>250
**31**	ii	β-OH	β-OH	=O	H	α-OH	R65	>250	>250
**32**	ii	=O	=O	=O	β-OAc	=O	R65	>250	>250
**33**	ii	β-OH	β-OH	=O	β-OH	=O	R65	>250	>250
**34**	ii	β-OH	=O	=O	β-OAc	=O	R65	>250	>250
**35**	ii	β-OH	=O	=O	H	=O	R65	>250	>250

*^a^* The activity was shown as IC_50_ value, which was the concentration (μM) of tested compound that resulted in 50% inhibition of cell growth. Results were expressed as the mean value of triplicate data points.

**Table 4:** Cell survival affected by Compounds 1-4, 8, 17, 20, 22 and 24

Compounds	IC50	compounds	IC50
**1**	42.0 ± 1.9	**17**	43.0 ± 0.3
**2**	36.5 ± 3.4	**20**	5.3 ± 0.8
**3**	4.9 ± 0.3	**22**	18.4 ± 3.2
**4**	21.7 ± 2.8	**24**	24.0 ± 2.5
**8**	14.6 ± 0.4		

**Figure 4:**
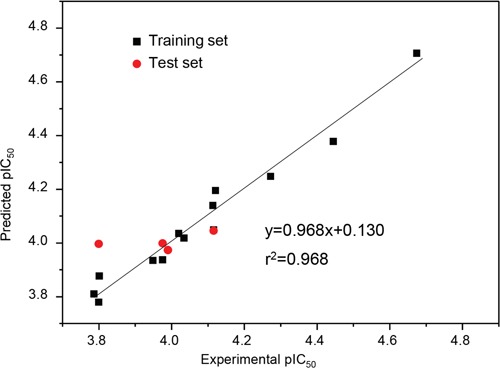
Experimental versus predicted breast carcinoma inhibitory activities of the training set and the test set The well agreement between predicted *p*IC_50_ value and experimental *p*IC_50_ value for both test sets and training sets indicated that this model was reliable in forecasting activity for *G. lucidum* triterpenoids.

**Figure 5:**
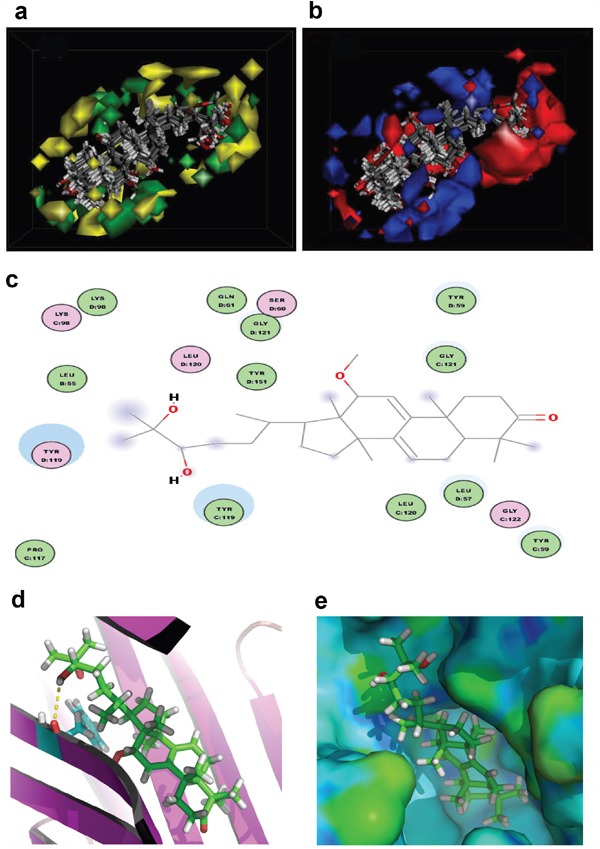
3D-QSAR model and docking analysis. a. 3D-QSAR model coefficients of triterpenoids from G lucidum on van der Waals grids. Green represents positive coefficients; yellow represents negative coefficients. **b**. 3D-QSAR model coefficients on electrostatic potential grids. Blue represents positive coefficients; red represents negative coefficients. **c**. 2D diagram of the interaction between compound 3 and the binding site of TNF-α. The H-bond (yellow dash) is displayed. **d**. 3D diagram of the interaction between compound 3 and the binding site of TNF-α. For clarity, only interacting residues are displayed. The H-bond (yellow dash) is displayed. **e**. The receptor surface model with compound 3.

3D-QSAR was then used to investigate the structure-activity relationship for inhibiting human breast cancer cells MDA-MB-231. Illustrated in Table 5, the training and test set of the 17 compounds (**1**-**9, 17, 19**-**24** and **28**) with accurate IC_50_ ranging from 21.2 to 163.5 μM was randomly selected for correlation analysis in due proportion that ratio of training set was 0.765, and ratio of test set was 0.235 by the Diverse Molecules method of Discovery Studio 3.1. The calculated *p*IC_50_ values ranged from 3.78 to 4.71. The correlation coefficient (*r*^2^) between the observed and predicted activity of the training set was found to be 0.968, whereas that of the test set was found to be 0.317, which proved that this QSAR model was acceptable. The predicted *p*IC_50_ values and residual errors of the 17 compounds analyzed using this QSAR model were listed in Table 5. A plot of the observed *p*IC_50_ versus the predicted data is provided in Figure 4, in which the plot of the actual IC_50_ versus the predicted values indicated that this model was reliable in forecasting activity for *G. lucidum* triterpenoids. Moreover, the molecules aligned with the *iso*-surfaces of the 3D-QSAR model coefficients on van der Waals grids (Figure 5a) and electrostatic potential grids (Figure 5b). It was widely accepted that a better inhibitor based on the 3D-QSAR model should have strong van der Waals attraction in the green areas and a polar group in the blue electrostatic potential areas (which were dominant close to the skeleton).

**Table 5:** Experimental and predicted inhibitory activities of 17 compounds by 3D-QSAR model

CLompounds	Experimental pIC_50_	Predicted pIC_50_	Residual error
1	4.27240	4.24780	0.0245988
2	4.44473	4.37745	0.0672809
3	4.67445	4.70636	-0.0319126
4	4.12084	4.19534	-0.0745045
5	3.94868	3.93447	0.0142062
6^a^	3.99007	3.97291	0.0171585
7	4.11364	4.13921	-0.0255712
8	4.01952	4.03475	-0.0152263
9^a^	3.79952	3.99636	-0.196836
17	4.03403	4.01796	0.0160717
19^a^	3.97562	3.99842	-0.022796
20	4.11556	4.04926	0.0663042
21	3.80137	3.87671	-0.0753358
22	3.97562	3.93744	0.0381821
23	3.78648	3.81034	-0.0238569
24^a^	4.11556	4.04527	0.0702946
28	3.79945	3.77969	0.0197632

*^a^* Compounds were selected as the test sets while the rest ones were in the training sets.

According to the modeling result provided in Figure 5a-5b, introducing slight bulk and lowly negative charged groups at C-25 or C-26 may elevate the activity of compound **1**. Meanwhile, introducing slightly bulk and low positive charged substitutes at C-7 and C-15 may increase the activity of compound **1**. Inversely, introducing bulk substitutes at C-1, C-2, C-3, C-22 and C-24 may decrease the activity of compound **1**. However, replacing C-1, C-2 and C-3 with negative charged moieties may increase the activity of compound **1**, while replacing C-19, C-23 and C-24 with low positive moieties may raise the activity of compound **1**. For compounds **9, 21** and **23**, the introduction of methyl group of steric hindrance at C-2 and negative *O* atom such as carbonyl group at C-7 to replace neutral *H* atom may decline their activity from the IC_50_ of 21.2 μM for compound **3** to about 160 μM for compounds **9, 21** and **23** (approximately 8 folds).

As depicted in Table 3, compound **3** showed the highest cytotoxicity, which suggests that compound **3** may exhibit the most potent affinity for its target. Since experimentally identifying and validating a target for a biological agent is time-consuming and costly, we used Pharmaceutical Target Seeker (PTS) [40] to predict potential targets of compound **3** and found that its most possible target is tumor necrosis factor α (TNF-α; PDB code: 2AZ5). To gain better understanding on the potency of the compound, we analyzed the interaction of compound **3** with TNF-α. The molecular docking was performed by inserting the compound into the binding site of TNF-α. All docking runs were applied by Discovery Studio. The binding interaction energy (-142.896 ± 50.365 kJ/mol) was predicted. Figure 5c-5e showed the binding mode of compound **3** interacting with 2AZ5 protein and the docking results revealed that the amino acid Tyr119 located in the binding pocket of the protein played vital role in the conformation with compound **3**, which were stabilized by one hydrogen bond. The hydrogen bond was formed relating to Tyr119, which connected to hydrogen atom of hydroxyl of compound **3** part with 2.8 °A. This molecular docking model suggests that compounds **3** may target TNF-α. TNF-α is an extraordinarily pleiotropic cytokine with a central role in immune homeostasis, inflammation, and host defense. TNF-α is a double-dealer. On one hand, TNF-α functions as an endogenous tumor promoter, because TNF-α stimulates cancer cell proliferation, invasion and metastasis. It also induces tumor angiogenesis. On the other hand, TNF-α functions as a cancer killer. Modulation of the activity of TNF-α will offer possibilities for cancer therapy. Since *Ganoderma lucidum* plays a central role in immune homeostasis, compound **3** may be developed as an agent for cancer immunotherapy.

## CONCLUSIONS

35 triterpenoids including 5 new compounds were isolated from the fruiting bodies of *Ganoderma lucidum*. The chemical structures of the new compounds were elucidated by spectroscopy. All of the compounds were assayed for their cytotoxic activity against the human breast carcinoma cell line MDA-MB-231 and hepatocellular carcinoma cell line HepG2, and the structure–activity relationships were revealed by 3D-QASR. Compound **3** showed the highest cytotoxic activity and it may target TNF-α. Our work may provide a guideline to design and optimize more effective inhibitors for human breast carcinoma based on the triterpenoids from *Ganoderma lucidum*. Our next task is to chemically synthesize this compound, confirm its activity, and explore its anticancer mechanism, and develop it as an agent for cancer immunotherapy.

## MATERIALS AND METHODS

### General experimental procedures

The ESI-MS spectra were recorded on a 6430 Triple Quad mass spectrometer (Agilent Technologies, Santa Clara, USA). The HR-ESI-MS spectra were obtained on a Micromass Q-TOF mass spectrometer (Waters Corporation, Milford, USA). 1D and 2D NMR spectra were measured on a Bruker AVANCE III 600 spectrometer. UV data were recorded using a JASCO V-550 UV/vis spectrometer (Jasco International Co., Ltd., Tokyo, Japan). IR data were recorded on a JASCO FT/IR-480 plus spectrometer (Jasco International Co., Ltd., Tokyo, Japan). Optical rotations were measured on a JASCO P1020 digital polarimeter (Jasco International Co., Ltd., Tokyo, Japan). Analytical HPLC was performed on an Agilent 1100 with an Agilent DAD spectrophotometer and a YMC-Pack Pro C18 (5 μm, 4.6 × 250 mm), while preparative HPLC was performed on an Shimadzu LC-20A spectrophotometer and a YMC-Pack Pro C18 column (5 μm, 20 × 250 mm). Normal phase silica gel (200–300 mesh, Qingdao Haiyang Chemical Co., Ltd.) and octadecylsilanized (ODS) silica gel (50 μm, YMC Ltd., Japan) were also used for column chromatography (CC).

### Fungi material

The quality of *Ganoderma lucidum* fruit body, collected in Dabie Moutain, Anhui, China, was inspected and analyzed by Yuewei Edible Fungi Technology Co. Ltd., Guangzhou, China. The voucher specimen (No. GL20151010) was deposited in State Key Laboratory of Applied Microbiology Southern China, Guangdong Institute of Microbiology.

### Extraction and isolation

The air-dried and powdered fruit bodies of *Ganoderma lucidum* (6 kg) were extracted with ethyl acetate (48 L × 2, v/v). The crude extract (130 g) was then incubated in chloroform (3 L × 3) to generate the total triterpenoids fraction (78 g). An aliquot of the chloroform extract (70 g) was applied to a silica gel column (200–300 mesh) eluted successively with CHCl_3_–MeOH (100:0, 98:2, 95:5, 90:10, 80:20, 70:30, 50:50 and 0:100) to obtain 8 fractions (Fr. 1∼ Fr. 8). Fr. 4 was subjected to a ODS silica gel column eluted with MeOH–H_2_O (30:70, 50:50, 60:40, 70:30, 80:20, 90:10 and 100:0) to afford 7 subfractions (Fr. 4.1∼ Fr. 4.7). Fr. 4.5 was further purified by preparative HPLC (MeOH–H_2_O, 80:20) to afford compounds **6, 1, 2** and **7**. Fr. 4.4 was further purified by preparative HPLC (MeOH–H_2_O, 70:30) to afford compounds **3, 4, 8, 9, 10, 11, 12** and **13**. Fr. 4.3 was purified by preparative HPLC (MeOH–H_2_O, 60:40) to produce compounds **14** and **15**. Fr. 3 was subjected to a ODS silica gel column eluted with MeOH–H_2_O (40:60, 50:50, 60:40, 70:30, 80:20, 90:10 and 100:0) to afford 7 subfractions (Fr. 3.1∼ Fr. 3.7). Fr. 3.5 was further purified by preparative HPLC (MeOH–H_2_O, 75:25) to produce compounds **16, 17, 18, 19, 20, 21, 5, 22, 23** and **24**. Fr. 3.4 was further purified by preparative HPLC (MeOH–H_2_O, 65:35) to afford compounds **29, 25, 30, 26, 27** and **28**. Fr. 3.3 was purified by preparative HPLC (MeOH–H_2_O, 55:45) to afford compounds **31, 32, 33, 34**, and **35**.

### Characterization of the new compounds

**Compound 1** (ganodermanontetrol): White powder; [α]_D_^25^ 13.4 (*c* = 0.20, CHCl_3_). UV (MeOH) λ_max_ 254.2 nm; IR (KBr) *v*_max_ 3393, 2945, 2858, 1709, 1658, 1459, 1415, 1375, 1261, 1095 cm^-1^; ESI-MS: m/z 999 [2M + Na] ^+^, 511 [M + Na] ^+^, m/z 487 [M - H] ^-^, 975 [2M - H] ^-^. HR-ESI-MS: m/z 487.3429 [M - H] ^-^ (cacld for C_30_H_47_O_5_, 487.3423); ^1^H-NMR (CDCl_3_, 600 MHz) and ^13^C-NMR (CDCl_3_, 150 MHz): see Table 1.

**Compound 2** (3*β*, 24, 25, 26-tetradroxy-7*α*-methoxy-8-ene-lanost-ol): White powder; [α]_D_^25^ 22.6 (*c* = 0.20, CHCl_3_). UV (MeOH) λmax 254.2 nm; IR (KBr) *v*_max_ 3387, 2940, 2862, 1713, 1645, 1450, 1418, 1375, 995 cm^-1^; 3387, 2925, 2854, 1722, 1656, 1601, 1507, 1361, 1290, 1255, 1171, 1069, 1022, 929; ESI-MS: m/z 1035 [2M + Na] ^+^, 529 [M + Na] ^+^, m/z 505 [M - H] ^-^, 1011 [2M - H] ^-^. HR-ESI-MS: m/z 505.3882 [M - H] ^-^ (cacld for C_31_H_53_O_5_, 505.3893); ^1^H-NMR (CDCl_3_, 600 MHz) and ^13^C-NMR (CDCl_3_, 150 MHz): see Table 1.

**Compound 3** (12*α*-methoxy-ganodermanondiol): White powder; [α]_D_^25^ 24.8 (*c* = 0.20, CHCl_3_). UV (MeOH) λmax 254.2 nm; IR (KBr) *v*_max_ 3355, 2920, 2851, 1720, 1650, 1450, 1278, 1012 cm^-1^; ESI-MS: m/z 995 [2M + Na] ^+^, 509 [M + Na] ^+^, 485 [M - H] ^-^, 971 [2M - H] ^-^. HR-ESI-MS: m/z 485.3642 [M - H] ^-^ (cacld for C_31_H_49_O_4_, 485.3631); ^1^H-NMR (CDCl_3_, 600 MHz) and ^13^C-NMR (CDCl_3_, 150 MHz): see Table 1.

**Compound 4** (15*β*-hydroxy-lucidumol A): White powder; [α]_D_^25^ 18.5 (*c* = 0.20, CHCl_3_). UV (MeOH) λ_max_ 254.2 nm; IR (KBr) *v*_max_ 3385, 2920, 2838, 1715, 1650, 1452, 1267, 1025 cm^-1^; ESI-MS: m/z 999 [2M + Na] ^+^, 511 [M + Na] ^+^, m/z 487 [M - H] ^-^, 975 [2M - H] ^-^. HR-ESI-MS: m/z 487.3435 [M - H] ^-^ (cacld for C_30_H_47_O_5_, 487.3423); ^1^H-NMR (CDCl_3_, 600 MHz) and ^13^C-NMR (CDCl_3_, 150 MHz): see Table 1.

**Compound 5** (15*α*-hydroxy-ganodermanontriol): White powder; [α]_D_^25^ 25.2 (*c* = 0.20, CHCl_3_). UV (MeOH) λmax 254.2 nm; IR (KBr) *v*_max_ 3389, 2925, 2835, 1724, 1655, 1455, 1258, 985 cm^-1^; ESI-MS: m/z 999 [2M + Na] ^+^, 511 [M + Na] ^+^, m/z 487 [M - H] ^-^, 975 [2M - H] ^-^. HR-ESI-MS: m/z 487.3438 [M - H] ^-^ (cacld for C_30_H_47_O_5_, 487.3423); ^1^H-NMR (CDCl_3_, 600 MHz) and ^13^C-NMR (CDCl_3_, 150 MHz): see Table 1.

### Viability and cell death assay

The cell culturing conditions, viability, and cell death assay was performed as described [41–43]. In brief, human breast carcinoma cell line MDA-MB-231 and hepatocellular carcinoma cell line HepG2 were used in the study. The cells were cultured in DMEM supplemented with 10%FBS, 100 U/mL penicillin/streptomycin at 37°C, 5%CO_2_ in an incubator (SANYO, MCO-18AIC). Cells (1 × 10^5^ cells/mL) were seeded in 24-well plates (500 μL/well). Four hours after inoculation, Compounds **1**-**35** were added individually into the cultured cells at different concentrations. After 48 h incubation, the cells were detached by trypsin, collected and analyzed by trypan blue staining for cell viability. Each experiment was repeated three times. Each treatment was performed with three replicates. Cell inhibition rate (IR) was expressed as follows:

IR (%) = (total cell number - living cell number)/total cell number×100%

IR and the corresponding concentrations of the compounds were inputed into SPSS and the Probit analysis was uesd for IC_50_ calculation. Data were expressed as mean ± SD (standard deviation).

### Cell survival assay

The survival assay was performed as described [44, 45]. MDA-MB-231 cells (5 × 10^4^ cells/well) were seeded in 24-well plates with 500 μL DMEM containing 10% FBS. 24-hour after cell inoculation, cells were washed gently with PBS twice and cultured in serum-free DMEM. The compounds **1**-**4, 8, 17, 19, 20, 22** and **24** were added to the wells at different concentrations. The medium used to dissolve compounds served as a control. After 24 h treatment, the cells were harvested, counted using trypan blue staining. The experiments of each compound were repeated three times. Each treatment contained three replicates. Cell inhibition rate (IR) was expressed as follows:

IR (%) = (total cell number - living cell number)/total cell number×100%

IR and the corresponding concentrations of the compounds were inputed into SPSS and the Probit analysis was uesd for IC_50_ calculation. Data were expressed as mean ± SD.

### QSAR model

A subset of 13 compounds was utilized as a training set for QSAR modeling using the procedure as described [46, 47]. Because it is essential to assess the predictive power of the resulting QSAR models on an external set of inhibitors, the remaining 4 molecules (ca. 25 % of the dataset) were employed as an external test subset for validating the QSAR models by the Diverse Molecules protocol in the Discovery Studio 3.1. The selected test compounds were compounds **6, 9, 19** and **24**.

The inhibitory effect of the compounds observed (IC_50_; μM) was changed to a negative logarithmic scale (*p*IC_50_; μM), and then used for subsequent QSAR analyses as a response variable.

In the Discovery Studio 3.1, the CHARMm force field was used and the electrostatic potential and the van der Waals potential were treated as separate terms. A +1e point charge was used as the electrostatic potential probe and the distance dependent dielectric constant was used to mimic the solvation effect. For the van der Waals potential, a carbon atom with a radius of 1.73 °A was used as a probe. The truncation for both steric and the electrostatic energies was set to 30 kcal/mol. Standard parameters were implemented in the Discovery Studio 3.1. A partial least-squares (PLS) model was built using energy grids as descriptors. QSAR models were built using the 3D-QSAR protocol of Discovery Studio 3.1.

### Target seeking and docking

For the molecular docking model, the three-dimensional X-ray structure of searched target acquired from the RCSB protein data bank (http://www.pdb.org) was selected as the template. All bound water and ligands were eliminated from the protein and the polar hydrogen was added to the proteins. The docking procedure was carried out using CDOCKER protocol for receptor-ligand interaction section of Discovery Studio [48]. Initially, the three-dimensional structures of the compound in this paper were built and energetically minimized by using MMFF94 with 5000 iterations and minimum RMS gradient of 0.10. Molecular docking of all compounds was then performed using the Discovery Studio as implemented through the graphical user interface CDOCKER protocol. CDOCKER is an implementation of a CHARMm based molecular docking tool using a rigid receptor.
